# Aldosterone in the brain and cognition: knowns and unknowns

**DOI:** 10.3389/fendo.2024.1456211

**Published:** 2024-11-01

**Authors:** Anna Nieckarz, Beata Graff, Michel Burnier, Anna B. Marcinkowska, Krzysztof Narkiewicz

**Affiliations:** ^1^ Department of Hypertension and Diabetology, Medical University of Gdańsk, Gdańsk, Poland; ^2^ Faculty of Biology and Medicine, University of Lausanne, Lausanne, Switzerland; ^3^ Applied Cognitive Neuroscience Lab, Department of Neurophysiology, Neuropsychology and Neuroinformatics, Medical University of Gdańsk, Gdańsk, Poland

**Keywords:** aldosterone, central nervous system, cognition, hypertension, primary

## Abstract

Mineralocorticoid receptors are expressed in several structures of the central nervous system, and aldosterone levels can be measured in the brain, although in smaller amounts than in plasma. Nevertheless, these amounts appear to be sufficient to elicit substantial clinical effects. Primary aldosteronism, characterized by high levels of plasma aldosterone, is one of the most common causes of secondary hypertension. In this context, high aldosterone levels may have both indirect and direct effects on the brain with a negative impact on several cerebral functions. Thus, chronic aldosterone excess has been associated with symptoms of anxiety and depression – two clinical entities themselves associated with cognitive deficits. Today, there is an increasing number of reports on the influence of aldosterone on the brain, but there is also a significant amount of uncertainty, such as the role of high aldosterone levels on cognitive functions and decline independently of blood pressure. In this mini review, we discuss the known and unknowns of the impact of aldosterone on the brain putting emphasis on cognitive functions.

## Introduction

Arterial hypertension is a disease that affects approximately 30% of adults worldwide (1, 2). In a significant number of patients, surpassing 10%, hypertension is a manifestation of one of at least 15 endocrine disorders leading to an excess or a deficit in the production of hormones contributing to the regulation of blood pressure (3). These disorders include primary aldosteronism, which is currently considered to be one of the most common causes of secondary hypertension, with an estimated prevalence ranging between 5% to 10% (4). Primary aldosteronism, also known as Conn’s syndrome, however, is not a homogenous disease; indeed, there are several forms of primary aldosteronism, all of them characterized by an excessive autonomous production of aldosterone. The two most common forms of aldosteronism are adrenal adenoma and adrenal hyperplasia, which can be uni- or bilateral, while other causes of aldosteronism, such as adrenal carcinoma or familial aldosteronism, are rather rare (5). Whereas primary aldosteronism was considered to be a disease affecting less than 1% of patients with hypertension some decades ago (6, 7), it is now recognized to be the cause of hypertension in about one out of 10 hypertensive patients (1). The main cause for this increase likely relies on the fact that, today, the diagnosis of primary aldosteronism is not limited to patients with hypertension and hypokalemia. Besides elevated blood pressure, the most characteristic clinical finding associated with primary aldosteronism is low plasma potassium level. However, hypokalemia is present in less than 40% of patients with this disorder, and most of the time the clinical presentation of aldosteronism is indistinguishable from primary hypertension (1). Therefore, it is now recommended to consider an hyperaldosteronism whenever proper blood pressure control is difficult to obtain or observed hypertension-mediated cardiovascular complications are more severe than expected (1). Indeed, studies have demonstrated that for the same level of blood pressure, patients with primary aldosteronism develop more severe target organ damage (1, 8, 9). In addition, studies have shown that the percentage of patients with primary hyperaldosteronism is greater in patients diagnosed with apparent drug-resistant hypertension and reaches up to 24% of these patients (4). Primary hyperaldosteronism is also the most frequent reason for secondary hypertension in middle-aged people (1), among whom nearly 50 years is the median age of diagnosis (10). Therefore, there is a trend to develop specific guidelines for the diagnosis of primary hyperaldosteronism (11). One interesting but very often overlooked clinical aspect of primary hyperaldosteronism is that anxiety and depression are more frequent in patients with hyperaldosteronism than in patients with essential hypertension (12). These complications may simply be due to uncontrolled hypertension but there is now increasing evidence that it might be a direct consequence of the aldosterone excess via endocrine effects on the brain as will be discussed below.

## Non-renal effects of aldosterone

The main role of aldosterone is to increase sodium reabsorption and to excrete potassium through the activation of mineralocorticoid receptors and the synthesis of epithelial sodium channels located in the distal convoluted tubule of the kidneys (13). However, there is now strong evidence supporting the presence of mineralocorticoid receptors at non-epithelial sites in the heart, and vasculature but also in the brain. Mineralocorticoid receptors are part of the transcription factor family activated by steroids. Upon activation through ligand-binding domain, the MR is translocated to the nucleus, where it functions as a transcription factor. Consequently, the effects of aldosterone have traditionally been associated with the regulation of gene expression activating the genomic pathway (14, 15). Although, in mentioned tissues, aldosterone may act through rapid non-genomic signaling pathways, the rapid effects usually supporting the physiological and pathophysiological genomic effects of aldosterone or contributing to regulatory loops. Yet, non-genomic aldosterone signaling can also lead to long-lasting and persistent effects such as fibrosis in the vasculature and the heart, vascular stiffening, and endothelial dysfunction (16). Thus, aldosterone excess not only leads to complications such as arterial hypertension due to an increase in renal sodium reabsorption, but also contributes to the development of organ damage such as left ventricular hypertrophy, peripheral arterial disease, or chronic kidney disease (17). Therefore, hypertension and heart failure are not the only indications for mineralocorticoid receptor antagonists. Today, with the development of non-steroidal mineralocorticoid receptor antagonists, such as finerenone, the number of indications is increasing (18). The recent demonstration of the clinical benefits on mortality and morbidity of finerenone in patients with diabetic nephropathies open new avenues for the use of mineralocorticoid receptor antagonists in medicine (19, 20).

## Aldosterone and mineralocorticoid receptors in the brain

A deterioration of brain function due to direct and indirect effects of high aldosterone levels might also occur but, so far, less attention has been given to the impact of aldosterone on the brain. The concentration of aldosterone in the brain is low because aldosterone has a relatively poor penetration of the blood–brain barrier compared with other steroids. Yet, aldosterone concentration in the central nervous system [CNS] is proportional to its serum concentration (21). In addition, aldosterone appears to be synthesized within the CNS (21–23). However, the exact role of brain-synthesized aldosterone and the synthesis sites have yet to be fully explored (23). Mineralocorticoid receptors have also been located in the brain (23, 24). Indeed, the presence of mineralocorticoid receptors is currently documented in several CNS structures responsible for cognitive functions, such as the hippocampus, amygdala, prefrontal cortex, and brain vessels (25, 26). Apart from the presence of mineralocorticoid receptors, the expression of 11ß-hydroxysteroid dehydrogenase type 2 [HSD2] is also required for aldosterone to access to neurons since cortisol and corticosterone are stronger ligands for brain MR receptors. HSD2 converts active glucocorticoids into inactive cortisone which enables aldosterone to bind to the receptors (27–29). Specific aldosterone-sensitive neurons were found in the nucleus of the solitary tract of both rats and mice, and in the subcommissural organ and the ventrolateral subdivision of the ventromedial nucleus of the hypothalamus of the rats only (27, 30, 31). In mice, 11β-HSD2 is highly expressed in the fetal CNS and transiently in early postnatal-maturing regions, notably the cerebellum and the enzyme activity is shut off with terminal differentiation (32). The elevated presence of the enzyme provides protection to the developing organism against the detrimental effects of excessive glucocorticoids, such as an increased predisposition to hypertension in later life. Studies have also shown that blood-brain barrier is permeable to aldosterone where HSD2-positive neurons are present. Furthermore, axonal projections from aldosterone-sensitive cells to the brainstem and forebrain structures have been described and this finding may explain the possible influence of aldosterone on brain functions such as appetite, mood, arousal, motivation or cognition (27, 29–31). Nevertheless, whether humans do have aldosterone-sensitive HSD2 neurons or not, and where these neurons are localized, if they exist, is still not proven.

## Effects of aldosterone on the brain

Studies have demonstrated that even modest aldosterone concentrations in the CNS induce noticeable reactions of the cardiovascular system. Gómez-Sánchez et al. have reported that a direct infusion of a small amount of aldosterone into the CNS of rats – an amount that would not cause any clinical effect if infused into the peripheral circulatory system – increases arterial blood pressure (33, 34). Similarly, infusion of mineralocorticoid receptor antagonists into the CNS, in equally small dosages, lowered blood pressure.

In humans, aldosterone has a negative impact on the cerebral vasculature. When infused, aldosterone decreases blood flow velocity and vascular reactivity, hence, directly affecting cerebral hemodynamics. The study performed by Hajjar et al. (35), in which 47 patients with essential hypertension were examined, found that blood flow velocity and CO_2_ vasoreactivity were significantly lower in patients with high aldosterone levels. Moreover, they observed that in the group with the highest levels of plasma aldosterone, a proper blood pressure control induced the greatest improvement of executive functions as well as CO_2_ vasoreactivity (35). Females represented 60% of the participants, and the observed differences remained significant after considering gender as a covariate.

In another study, Rizzoni et al. (36) compared small resistant arteries of 30 subjects in search of evidence of a profibrotic vascular effect of aldosterone. The participants were divided into three groups: the first one included 13 patients (6 males, 7 females) with primary aldosteronism, the second one 7 patients (3 males, 4 females) with essential hypertension, and the third one included 10 normotensive controls (5 males, 5 females). By using micromyography to assess artery morphology, authors demonstrated that in patients with primary aldosteronism, fibrosis of the small vessels was more severe than in patients with primary hypertension. Taken together, these findings may suggest that aldosterone contributes to a deterioration in cerebral blood supply and, consequently, to alterations of brain functions observed in patients with primary hyperaldosteronism.

## Hypertension and cognitive functions

Describing the possible influence of aldosterone on cognitive functions, it is impossible not to emphasize the impact of hypertension itself on this issue, as in a prominent percentage of cases the cause of abnormal blood pressure values may be an excess of aldosterone, and the most common clinical effect of aldosterone excess is hypertension. In humans, a persistently elevated blood pressure is known as a major risk factor of cognitive decline (37, 38). Indeed, untreated or improperly treated hypertension has been shown to damage both the cerebral vessels (39, 40) and blood–brain barrier (41), causing alterations in the functions and structures of the brain (40, 42). White matter lesions, particularly in the frontal cortex, are the most frequently observed brain abnormalities associated with hypertension (43). Other possible cerebral damages induced by hypertension include cerebral tissue atrophy, microinfarcts, microbleeds, interference with neuronal networks, reduced cerebral blood flow, or diminished neural clearance (44, 45). In middle-aged hypertensive patients, the impact of hypertension might manifests as a mild cognitive impairment (46). In older patients, uncontrolled hypertension significantly increases the risk of dementia (37). Therefore, further research is needed to fully understand the relationship between hypertension and cognitive impairment. Notably, increasing evidence suggests that blood pressure values may not be the primary cause of cognitive deterioration; rather, blood pressure variability may play a more significant role. Melgarejo et al. demonstrated that long-term changes in blood pressure variability, as measured by 24-hour ambulatory blood pressure monitoring, were associated with poorer outcomes in cognitive assessments, whereas increases in blood pressure levels did not produce similar results (47). In the sample of 437 participants studied, 67% were women, and the authors did not report any sex differences.

The term “cognitive functions” is understood to include processes such as memory and learning, attention, perception and visuospatial processes, and executive functions (48). The term “mild cognitive impairment” describes minor deficiencies in the processes mentioned above; the impairment of the quality of daily functioning is unnoticeable for the patient and for many clinicians. However, this deficiency is larger than it would be due to a senescence of the organism only (49).

## Aldosterone and cognitive functions

The renin-angiotensin-aldosterone system [RAAS] plays an important role in the regulation of several cerebral functions as reviewed by Cosarderelioglu et al. (50). Thus, studies carried out on animals have brought evidence that continuous activation of the RAAS can cause neuroinflammation, oxidative stress, endothelial dysfunction, microglial polarization, and alterations in neurotransmitter secretion in the brain (51). These effects can be mediated by several components of the RAAS including angiotensin II, but also angiotensin III and IV, and to a certain degree, aldosterone. When associated with decreases in brain perfusion, these effects of the RAAS may lead or contribute to a progressive decline in cognitive functions but also to the development of cerebral diseases such as Alzheimer disease (35, 52). As has been indicated earlier, mineralocorticoid receptors are present in various areas of the brain, including vascular endothelial cells. Dinh et al. demonstrated that aldosterone, acting through mineralocorticoid receptors in endothelial cells, increases superoxide levels and chemokine expression leading to oxidative stress and inflammation in the brain tissue (53).

It is now well-established that the glycocalyx represents the innermost structure of blood vessels. The structure consists of both components adsorbed from the bloodstream and elements attached to the cell surface, including proteoglycans and sialoproteins. The proper functioning of the endothelium is critically dependent on an intact and functional glycocalyx. Recent evidence suggests that stimulation of mineralocorticoid receptors may also contribute to glycocalyx damage, further impairing endothelial function (54). Overall, the aforementioned processes may result in cerebrovascular alterations, including cerebrovascular disease, stroke, or cognitive decline, independently of blood pressure levels.

### Animal studies

Inaba et al. evaluated cognitive functioning (learning in shuttle avoidance test), cerebral blood flow, and brain oxidative stress in wild-type male mice and transgenic male mice overexpressing the human renin and angiotensinogen genes (52). In their study, a decline in cognitive function was observed at an earlier stage, accompanied by a significant reduction in cerebral blood flow and a greater increase in oxidative stress in transgenic mice (52). In addition, an intriguing observation was that transgenic mice were unable to achieve the learning ability level shown by wild-type mice even before the cognitive decline started. Conversely, the study conducted by Manschot et al. on male rats showed that blockade of the RAAS resulted in increased blood perfusion through brain structures such as the hippocampus (55). Better hippocampal perfusion should have a positive impact on learning and verbal and spatial memory, which enable individual to comprehend and recollect spatial connections between different objects, as well as among objects and surroundings. One major limitation of these studies is that they do not analyze the specific role of each potential mediators of the RAAS cascade. Thus, one cannot assess whether the observed effects are mediated by blockade of angiotensin or aldosterone effects.

### Human studies

Today, it is well recognized that the higher the blood pressure the greater the risk of cognitive decline and dementia (56). Patients with primary hyperaldosteronism show significantly higher scores for depression and anxiety compared to the general population, and cognitive alterations are prevalent in depression (57). Therefore, high aldosterone levels may contribute to the development of cognitive dysfunction and depression. Interestingly, Yagi et al. additionally identified high plasma aldosterone levels as a risk factor for cognitive decline in patients with essential hypertension (22). Moreover, in a recent analysis of a large group of hypertensive Chinese patients (1094 participants, 38% females), a positive association between the level of circulating aldosterone and the presence of white matter lesions has been reported with no sex differences mentioned (58). These data would support the hypothesis of a relevant contribution of aldosterone to the development of cognitive dysfunction in patients with hypertension but perhaps partially independently of blood pressure. In this respect, it is interesting to note that the male and female children of Finnish women who electively consumed substantial amounts of liquorice (an inhibitor of 11β-HSD2), during pregnancy show a significant reduction in IQ when aged 8 and 12 and a significant increase in attention disorders, sleep disturbances and psychiatric outcomes, suggesting that feto-placental 11β-HSD2 inhibition during pregnancy impacts human brain function later in the life of children (59, 60).

Several studies indirectly suggest that aldosterone may be connected with cognitive deterioration in patients with arterial hypertension (35, 61, 62). In the study performed by Yagi et al. among people with primary hypertension, a subgroup of participants with higher plasma aldosterone concentration (but still within normal limits) received worse results in the Mini-Mental State Examination [MMSE] evaluating their cognitive functions (61). Moreover, these patients improved their test results after 6 months of mineralocorticoid receptor antagonist therapy. The study included 68 participants, 60% of whom were female. Apart from aldosterone concentrations, only age and stroke in the past medical history had an impact on cognition, suggesting that the effect of aldosterone is in part independent of blood pressure. This might indicate that aldosterone indeed increases the risk of both cardiovascular complications and progression of cognitive impairment (61). However, in the conducted study, only seven out of 68 enrolled participants were treated with a mineralocorticoid receptor antagonist. Furthermore, the MMSE is certainly a common tool to assess cognitive functions, but it serves only as a screening test and as such is insufficient. Hence, to perform a complete assessment of cognitive functioning, it is recommended to make use of a greater set of neuropsychological tests.

In contrast, Sen et al. have found that both the quality of blood pressure control and plasma angiotensin II concentrations had an impact on cognitive functions, whereas no significant correlation was found between plasma aldosterone concentration and the standardized MMSE results achieved by hypertensive patients (63). In this study, 41 people (63% females) treated for hypertension were examined, among them, none was treated with a mineralocorticoid receptor antagonist. There was no indication for the possible inclusion of patients with hyperaldosteronism. The main conclusion was that blood pressure needs to be under control to prevent cognitive impairment. Furthermore, Sen et al. admitted that their observations might have been affected by the selection of the patients, the low number of participants, and the study design (63). Thus, no unequivocal conclusion about the role of aldosterone on cognition could be derived from this study. Furthermore, MMSE was the only test utilized to assess cognitive impairment.

**Figure 1:**
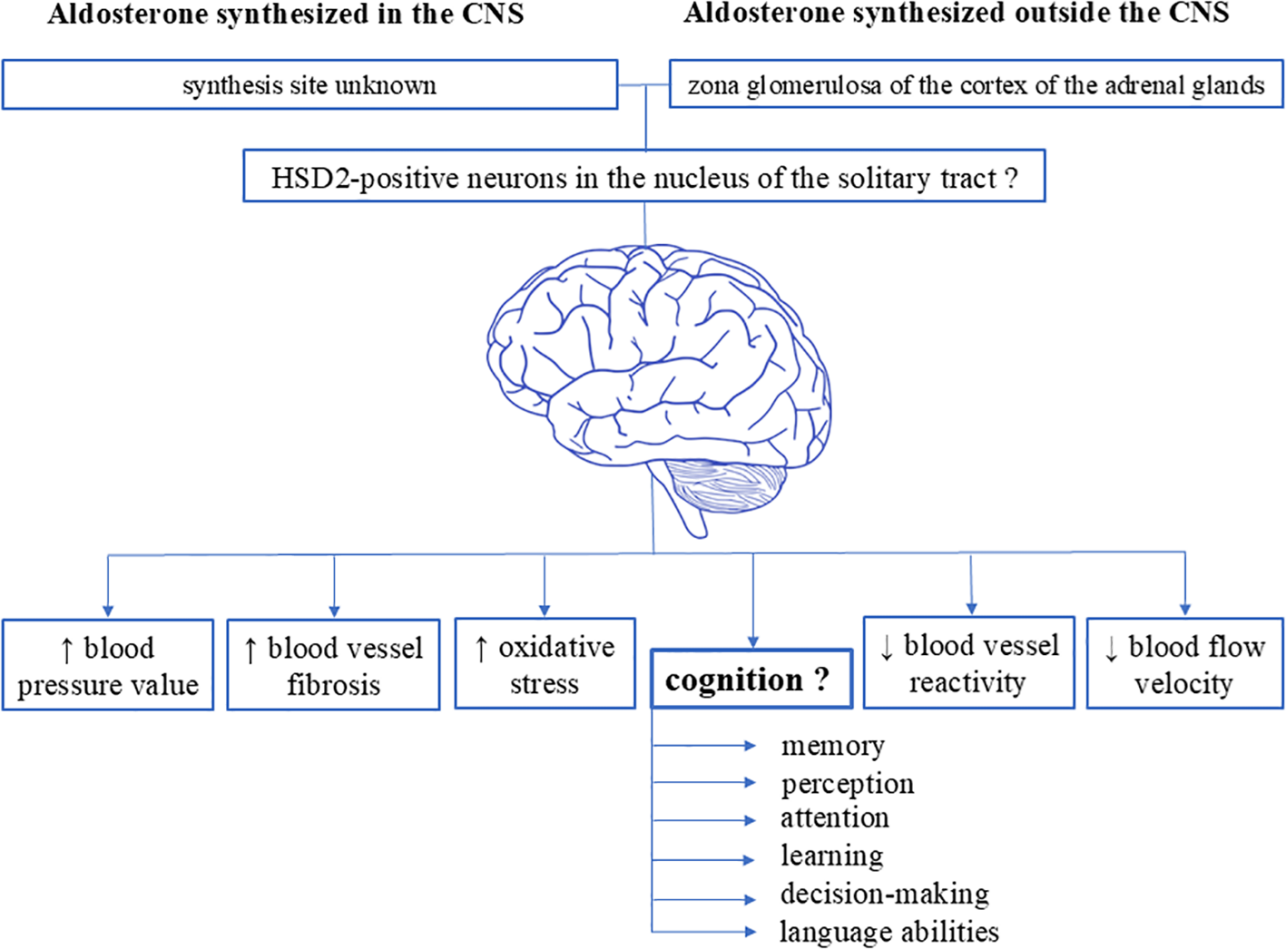
Schematic representation of the various effects of aldosterone on the brain. Existing data suggest the presence of specific aldosterone-sensitive neurons (characterized by the presence of 11ß-hydroxysteroid dehydrogenase type 2) in the central nervous system. Evidence indicate that these neurons are localized in the nucleus of the solitary tract in the medulla oblongata within the brainstem of rodents (both rats and mice), as well as in the subcommissural organ and the ventrolateral subdivision of the ventromedial nucleus of the hypothalamus in rats. However, it remains unknown whether humans possess aldosterone-sensitive HSD2 neurons, where these neurons might be localized, the exact synthesis sites of brain-synthesized aldosterone, and the impact of aldosterone on cognitive functions. CNS, central nervous system.

A recent study carried out by Engler et al. closely assessed the impact of aldosterone on cognition in patients with high aldosterone levels (57). To this purpose, authors examined 19 patients (53% females) newly diagnosed with primary hyperaldosteronism and used several psychiatric and neuropsychological scales to evaluate depression, anxiety, quality of life, quality of sleep, and cognitive functions such as selective attention, verbal memory, executive abilities, abstract reasoning and processing speed. Although symptoms of depression and anxiety, together with reduction in quality of life, were found in their patients, no specific effect of chronically elevated aldosterone levels was found on the cognitive function tests (57). However, the small number and younger age of the patients were the main limitations of this study, even though the neuropsychological assessments were extensive. Definitively, additional clinical studies of this type with comprehensive tests of cognitive functions are needed to explore the clinical impact of high aldosterone levels on cognition (**Figure 1**).

Nevertheless, it is worth noting that mentioned research suggests that aldosterone levels and their effects on cognition and cardiovascular function may differ between sexes. While chronic aldosterone excess did not significantly impact cognitive function overall in younger patients with primary aldosteronism, sex-specific correlations were observed between cognitive performance and anxiety, depression, and sleep disturbances (57). Gender disparities were also described by Shukri et al. (64) Their study provides evidence that females exhibit greater salt sensitivity of blood pressure, heightened aldosterone responses to stimuli, and may benefit more from mineralocorticoid receptors blockade therapy than males.

Of note, there is now increasing evidence from experimental as well as human studies that aldosterone has a causal role in stress-related disorders and may have an important role in the pathophysiology of depression (65). Thus, a positive correlation between aldosterone levels and the severity of depression has been reported and symptoms of depression, anxiety and somatization causing a reduced quality of life are frequently reported in patients with primary hyperaldosteronism. Evidence of central hyperactivation of aldosterone-sensitive mineralocorticoid receptors is observed in patients with depression, especially in those with atypical depression. Moreover, elevated aldosterone levels in individuals with depression have been linked to structural brain changes, including enlarged ventricles, compression of the corpus callosum, and alterations in the choroid plexus. These neuroanatomical changes are further correlated with poorer treatment outcomes (66).

In addition, further studies should be conducted on the pathogenesis of cognitive impairments associated with the global activity of the RAAS in the brain. Thus, there is a presumption that an angiotensinogen [AGT] gene polymorphism might be one of the reasons for discrepancies in the results of previously conducted studies investigating the role of the RAAS on cognition. The AGT gene encodes angiotensinogen, and some polymorphisms lead to increased activity of the RAAS in the Caucasian population (67). One research project has investigated the impact of polymorphisms in key RAAS genes on the association between angiotensin converting enzyme inhibitor exposure and global and executive cognitive functions. Analyses were performed on 3075 participants (52% females) enrolled in a prospective community-based study of well-functioning cognitively intact elderly participants (70-79 years of age) recruited between 1997 and 1998. Participants were recruited from a random sample of Caucasian and African American Medicare-eligible adults living in two American cities (Pittsburgh and Memphis). The observation period was 8 years, and cognitive functions were assessed by three cognitive tests: the MMSE, Executive Clock Drawing test-1, and the Digit Symbol Substitution test. The results of these analyses suggest that exposure to angiotensin converting enzyme inhibitors is protective against executive function decline only in individuals who have the AA genotype of the 6AG polymorphism or the CC genotype of the M235T polymorphism in the AGT gene. The AG or GG genotypes of the 6AG polymorphism and CT genotype of the M235T polymorphism in the AGT gene were associated with greater declines in Executive Clock Drawing test-1 scores only if they were not exposed to angiotensin converting enzyme inhibitors. These associations were significant only in Caucasian adults. These data suggest that polymorphisms of the RAAS may play a role in cognitive functions in some but not all patients. However, these data do not provide any evidence on the role of aldosterone. Finally, these results would need to be verified in further clinical studies involving patients with different genotypes.

## Conclusions

Although it is well known that high blood pressure affects cognitive functions and that primary hyperaldosteronism leads to more cardiovascular and cerebral complications than primary hypertension, the influence of aldosterone on the human CNS is not yet thoroughly known. The number of studies that assessed the impact of aldosterone on cognition is small and, in most cases, the groups of participants were sparse, and results were ambiguous. Furthermore, most studies were conducted on patients with essential hypertension and not with primary hyperaldosteronism. Thus, even though plasma aldosterone levels were different, they were often still within laboratory norms in most studies. In addition, conclusions obtained from the studies performed on humans are primarily indirect. Nonetheless, there is some good evidence that aldosterone per se has a negative influence on cerebral vasculature and also directly on the brain. Therefore, there is a need to reinforce both the experimental and clinical research in that field, as the application of a proper treatments, such as selective mineralocorticoid receptor antagonists, may prevent the development of complications or reduce the present ones in patients with primary hyperaldosteronism. Preliminary experimental reports (68, 69) showing an improvement in cognitive functions in mice treated with mineralocorticoid receptor antagonists might set the stage for further experimental and clinical investigations (70). Moreover, today, several new drugs are being developed for the treatment of hypertension that will interfere with aldosterone actions such as non-steroidal mineralocorticoid receptor antagonists, centrally acting aminopeptidase inhibitors or aldosterone synthase inhibitors (71). Whether these new therapeutic approaches will improve our ability to prevent the progressive cognitive decline observed in hypertension definitively will have to be investigated.
